# Does Breastfeeding Small for Gestational Age Neonates Promote a Healthier Growth Pattern? A Narrative Review

**DOI:** 10.3390/children12091227

**Published:** 2025-09-13

**Authors:** Natalia Atzemoglou, Nikolaos P. Tzavellas, Niki Dermitzaki, Maria Baltogianni, Foteini Balomenou, Anastasios Serbis, Vasileios Giapros

**Affiliations:** 1 Neonatal Intensive Care Unit, University Hospital of Ioannina, 45500 Ioannina, Greece; n.tzavellas@uoi.gr (N.P.T.); n.dermitzaki@uoi.gr (N.D.); mbalt@doctors.org.uk (M.B.); f.balomenou@uoi.gr (F.B.); 2 Department of Pediatrics, University Hospital of Ioannina, 45500 Ioannina, Greece; aserbis@uoi.gr

**Keywords:** breastfeeding, growth, human milk, obesity, preterm infant, small for gestational age

## Abstract

**Background**: Small for gestational age neonates represent a population at risk of growth failure or deviant growth patterns and long-term metabolic complications. Breastfeeding has been identified as a critical factor in promoting healthier growth and long-term metabolic health in both full-term and preterm appropriate for gestational age infants, but similar studies in small for gestational age infants are limited. The aim of this narrative review is to assess the impact of breastfeeding on growth and body composition in small for gestational age neonates. **Methods**: The PubMed and Google Scholar databases were screened for the relevant literature. The following terms, were used: “low birth weight”, “in utero growth restriction”, “small for gestational age”, “human milk”, and “growth”. The initial screening identified 57 relevant studies. Thirteen of them fulfilled the eligibility criteria and were included in this narrative review. **Results**: In preterm small for gestational age neonates, human milk nutrition was associated with healthier catch-up growth without excessive fat accumulation. Fortification strategies were associated with enhanced growth outcomes without increased incidence of neonatal morbidities. In the context of full-term, small for gestational age neonates, exclusive breastfeeding has been demonstrated to be associated with healthy catch-up growth. Furthermore, human milk nutrition has been shown to mitigate the predisposition of these children to obesity and cardiometabolic complications. **Conclusions**: According to the limited extant literature, human milk feeding has been identified as a potentially protective factor for small for gestational age neonates, promoting healthier growth patterns and long-term cardiometabolic health. However, larger prospective studies are needed to evaluate human milk feeding and human milk fortification in association with growth and long-term outcomes in small for gestational age infants.

## 1. Introduction

The first 1000 days of life, from conception to the second year, represent a critical period of rapid growth and organ development, characterized by high nutritional requirements. In addition to the heightened requirements for ensuring optimal growth and organ maturation, this period is distinguished by an increased vulnerability to external stimuli [1]. Undernutrition during this critical phase can result in long-term, irreversible consequences, including impaired linear growth and potential damage to the developing central nervous system. Moreover, the extant literature supports the notion that undernutrition and overnutrition during the early stages of development are associated with cardiometabolic consequences in later childhood and adulthood [1,2].

Particularly challenged in achieving optimal nutrition during this vulnerable period is the group of preterm neonates (neonates born before the 37th week of gestation), particularly very preterm neonates with a gestational age of less than 32 weeks, in which the critical third trimester of pregnancy is missed [3,4]. Preterm neonates and neonates with impaired prenatal nutrition (growth-restricted) due to reduced nutrient supply are highly susceptible to adverse metabolic and neurodevelopmental consequences due to antenatal undernutrition. It is well documented that infants born preterm, with growth restriction, and those with a low birth weight (i.e., <2500 g), including those infants who are small for gestational age, are prone to excessive postnatal growth and its consequences [3,5]. This is of particular concern in cases of very low birth weight (i.e., <1500 g) and extremely low birth weight (i.e., <1000 g) preterm neonates, especially those who are small for their gestational age [6]. This has been shown to increase the risk of obesity and metabolic complications later in life.

### 1.1. Small for Gestational Age (SGA)

According to the World Health Organization (WHO), a neonate is defined as small for gestational age (SGA) if its birth weight is below the 10th percentile for its gestational age and sex [7]. The occurrence of SGA may result from a pathological process or may reflect constitutionally small neonates [8,9]. A term that should not be confused with SGA is IUGR (intrauterine growth restriction), which refers to a fetus that is unable to achieve its growth potential due to an adverse intrauterine environment, as determined by serial fetal ultrasound measurements [9,10]. SGA neonates may be born either at term or preterm. Regardless of the gestational age, SGA infants represent a high-risk group for perinatal morbidity, developmental delays, and long-term adverse outcomes [5,11,12,13].

Firstly, SGA newborns have a higher probability of complications such as sepsis and the need for mechanical ventilation, which are directly linked to poorer neurodevelopmental outcomes [14,15,16]. In the long term, SGA newborns, particularly those who do not receive breast milk, are more prone to infections, including gastroenteritis and respiratory infections [17,18]. Moreover, SGA neonates are at an increased risk of chronic pathologies such as cardiovascular disease, diabetes, hypertension, and obesity [5,12,19]. Longitudinal studies of SGA individuals in childhood suggest that cognitive developmental challenges primarily manifest as poor academic performance in childhood. However, these differences may be less evident in adulthood [20,21].

The above outcomes are at least partly associated with the rapid postnatal growth catch-up that occurs during the first postnatal months. A substantial body of the literature supports the association between accelerated postnatal growth in SGA neonates and a series of adverse metabolic outcomes, including the increased risk of obesity, lower insulin sensitivity, reduced high-density lipoprotein (HDL) cholesterol, elevated triglyceride levels, and early markers of atherosclerosis [22,23,24,25,26]. Thus, postnatal growth patterns in these populations appear to be crucial in both the short and long term.

### 1.2. Human Milk Composition

Human milk is a complex biological fluid that contains essential nutrients and functional components, including essential long-chain fatty acids, complex oligosaccharides, nucleotides, bioactive signaling proteins, and hormones [27]. The nutrient composition of human milk is not constant but dynamic, depending on various parameters, including maternal diet, gestational and postnatal age, and environmental factors [27,28]. Human milk is, in general, composed of 87–88% water, 7% carbohydrates, 3.8% lipids, and 1% proteins [27].

The most abundant carbohydrate in human milk is lactose, and unlike the other nutrients, its concentration remains constant throughout lactation, thus maintaining a relatively constant osmotic pressure of human milk [27]. Human milk oligosaccharides (HMOs) represent the second most abundant carbohydrate in human milk and have been shown to be bioactive components, exerting a prebiotic role in the intestinal microbiota and displaying inflammatory and immunomodulatory properties [29]. Lipids in human milk are the primary source of energy. They also have a crucial role in central nervous system development, for inflammatory responses, digestion, membrane composition, the carrying of lipid-soluble vitamins, and are precursors of eicosanoids [28,30]. Except for vitamins K and D, human milk contains sufficient vitamins [28].

Human milk also contains a variety of hormones which aim to promote infant growth and development. These include progesterone, thyroid-stimulating hormones, glucocorticoids, and metabolic hormones [31]. Growth factors represent a class of bioactive compounds present in human milk that promote cell development and differentiation in various tissues and organs of the organism, including the central nervous system, gastrointestinal tract, and vasculature [32]. The concentration of these factors is elevated in the early postnatal period and subsequently declines over time [31,32]. The growth factors present in human milk include, among others, the epidermal growth factor, vascular endothelial growth factor, insulin growth factor, and granulocyte colony-stimulating growth factor [27,31].

A variety of bioactive factors with immunological and anti-inflammatory properties are present in human milk, particularly in the colostrum [33,34]. Secretory immunoglobulin A (sIgA), the most abundant immunoglobulin in human milk, primarily functions to enhance mucosal defense by binding to pathogens and preventing their adherence and epithelium penetration [35]. A variety of pro-inflammatory and anti-inflammatory cytokines are present in human milk, with a regulatory role in immune response [36]. Other compounds of human milk with immunological properties are lactoferrin, which are present in high concentrations in the colostrum, lysozyme, lactadherin, and alpha-lactalbumin [31].

The composition of human milk derived from preterm mothers has been found to differ compared to full term. A systematic review and meta-analysis of 41 studies reported significantly higher protein content in the preterm milk during the first postnatal days. The difference was progressively narrowed following the third day of life, and by the tenth week, no difference was found. Moreover, significantly lower levels of lactose were observed in preterm milk. No significant difference regarding fat content was reported [37]. Concerning the bioactive components of preterm milk, variations have also been reported in comparison to full-term milk [38]. Levels of sIgA are significantly higher in preterm milk, and a slower postnatal decline has been observed [38,39]. Regarding cytokines, it has been reported that the highest concentration is observed between 30 weeks of gestation and full term [40]. Higher concentrations of the epidermal growth factor and the transforming growth factor have been reported in preterm milk [38,40].

Human milk is the optimal source of nutrition for preterm neonates, as it provides essential nutrients and immune factors that support the development of their immature immune system [38]. However, the high nutritional requirements of very preterm neonates cannot be addressed by unfortified human milk. The fortification of human milk with nutrients, including protein, calcium, and phosphate is a common practice to ensure optimal growth [41]. In cases in which the mother’s own milk is not available, fortified, pasteurized, human-donor milk is considered the second most beneficial source of nutrition for preterm neonates [42,43]. As this milk is typically derived from mothers of full-term or more mature neonates, the content of their milk in micro- and macronutrients differs from that of preterm milk. It has been documented that there is a reduced level of protein, sodium, potassium, and chloride [44]. However, the donor milk retains the immunological properties and oligosaccharide content of human milk [43].

### 1.3. Infant Formula Composition

As previously described, human milk is a complex of nutritive and bioactive substances. Infant formulas have been produced as substitutes for human milk in cases where it is unavailable. The primary base for infant formula development is bovine milk, which is processed to resemble human milk [45].

Bovine milk has higher protein content compared to human milk, even following processing; the protein content is 1.3–1.5 g/100 mL. A high protein intake is associated with rapid weight gain and a potentially increased risk of obesity. Infant formula has lower lactose and higher casein compared to human milk. Infant formulas are supplied with the essential fatty acids; however, the arachidonic acid and docosahexaenoic acid are present in lower concentrations. Fortification of bovine milk with vitamins and minerals ensures adequate intake [45,46]. To address the increased nutritional requirements of very preterm and very low birth weight neonates, preterm infant formula has been formulated to contain elevated levels of protein, minerals (including calcium and phosphorus), medium-chain triglycerides (MCTs), and energy [6,47]. Human milk provides a range of bioactive compounds that support the immature immune system and promote growth and development. Supplementation of the bovine milk with lactoferrin, human milk oligosaccharides, prebiotics, and probiotics aims to resemble the valuable effects of human milk [45].

### 1.4. Breastfeeding

Human milk is regarded as the optimal nutrition for both term and preterm neonates and infants [48]. It fulfills all the nutritional requirements and contains all the essential nutrients and functional components needed in full-term infants up to the sixth postnatal month [48,49]. However, in cases of very preterm and very low birth weight neonates, human milk should be fortified with nutrients to ensure that the high nutritional needs of this population are met, and optimal growth is supported [41,50]. The WHO recommends that infants should be exclusively breastfed for the first six months of life and that supplementary feeding should be initiated thereafter, while breastfeeding is continued until the age of two years or beyond [51].

Human milk feeding has been shown to be associated with various short- and long-term benefits and is a key factor in maintaining health and promoting growth and cognitive development [48,52,53]. The benefits of breastfeeding are attributable to two primary mechanisms: the composition of the milk and the physical contact between the mother and infant during breastfeeding [27,28,54].

Breastfeeding has numerous benefits for full-term neonates [48]. Exclusive breastfeeding for three to four months has been associated with the reduced risk for atopic disease [55]. Breastfeeding has been shown to significantly reduce the incidence of gastrointestinal and respiratory infections during infancy [52,53]. As has been demonstrated, infants who are exclusively breastfed for four months or more exhibit a lower incidence of lower respiratory infections by more than 70% [48]. Furthermore, infants who are exclusively breastfed for more than six months show a fourfold decrease in pneumonia incidence compared to those breastfed for four to six months [56]. It has also been demonstrated that cognitive development is beneficially impacted by prolonged and exclusive breastfeeding [57]. Breastfeeding is associated with a lower risk of obesity, diabetes, and cardiovascular disease in adolescence and adulthood [48,58].

In addition to the previously mentioned short- and long-term benefits of human milk, preterm neonates, the most vulnerable neonatal population, gain some additional benefits [59]. It has been demonstrated that the incidence of several complications associated with prematurity, including necrotizing enterocolitis, retinopathy of prematurity, and bronchopulmonary dysplasia, is significantly decreased in neonates fed human milk [38,59,60].

A substantial body of research has been conducted on the potential benefits of breastfeeding in full-term and preterm infants. However, the primary focus of the research is on neonates with a weight appropriate for gestational age (AGA). Concerning the subject of the SGA, the evidence regarding the consequences of feeding practices is inconclusive. This is due to the fact that SGA neonates are less likely to initiate and continue breastfeeding than AGA neonates [61]. The nutritional management of preterm SGA neonates, concerning both early postnatal and later in life growth, represents a particularly complex challenge in the fields of neonatal and pediatric care. These infants, already compromised by intrauterine growth restrictions and premature birth, present a unique dilemma where immediate nutritional interventions must balance short-term growth requirements against long-term developmental optimization [62,63].

A deeper understanding of the role of breastfeeding in SGA infants regarding their early and later growth patterns will help to enhance outcomes in this vulnerable population, including term and premature SGA infants, who may have special dietary needs due to their low birth weight. The primary aim of this narrative review is to provide an overview of the extant literature regarding the beneficial effects of human milk nutrition on the growth of SGA infants. A secondary aim is to assess the potential long-term metabolic benefits of human milk in this vulnerable population.

## 2. Methods

**Figure 1:**
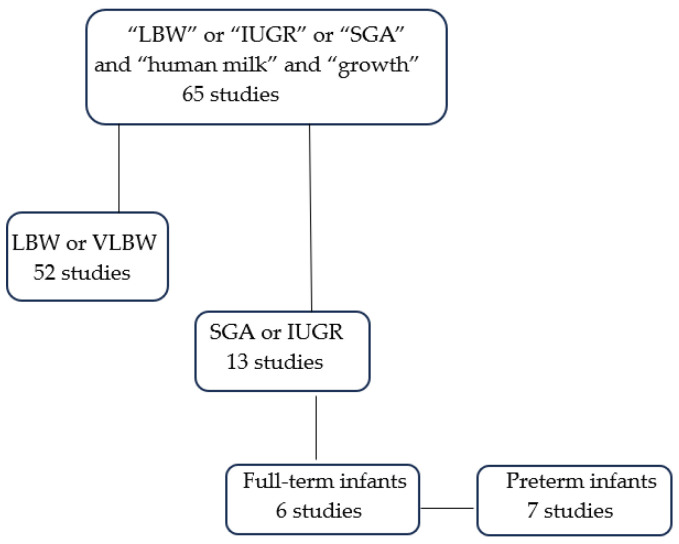
Flow diagram of the literature search.

A comprehensive search was conducted on the PubMed and Google Scholar databases to identify relevant studies on the impact of breastfeeding on the growth of both full-term and preterm SGA neonates. The following terms were used: “low birth weight”, “in utero growth restriction”, “small for gestational age”, “human milk”, and “growth”, up to June 2025. The titles and abstracts of the retrieved articles were scanned for relevance. We also reviewed the reference lists of the retrieved articles to identify other relevant articles that could have been missed in the initial search. Both retrospective and prospective cohort studies were included. Non-original articles and studies not published in English were excluded. Studies evaluating the effects of human milk on the growth outcomes of low birth weight infants that are not SGA were also excluded. Finally, 13 articles were included in this narrative review (Figure 1).

## 3. Results

A total of 13 studies, which evaluated the effects of human milk on growth patterns of preterm and full-term SGA neonates, were retrieved.

### 3.1. Preterm SGA Neonates and Breastfeeding

Seven studies that evaluated the growth patterns of human milk-fed preterm SGA neonates were included (Table 1) [64,65,66,67,68,69,70]. Significant heterogeneity was observed among the studies with regard to the study groups. The feeding practices differ among studies regarding the duration of human milk nutrition, the fortification, and the use of mother’s own milk (MOM) or MOM and donor milk. All studies evaluated growth with anthropometric measurements at hospital discharge, and three studies evaluated longitudinal growth outcomes (at the age of six months to two years) [67,69,70]. In one study, children were additionally evaluated with serum glucose, non-fasting insulin, and X-ray absorptiometry [65].

### 3.2. Full-Term SGA Neonates and Breastfeeding

Six studies that investigated the effect of human milk on the growth of full-term neonates were included (Table 2) [71,72,73,74,75,76]. Significant heterogeneity regarding the study groups, the duration of breastfeeding, and the age at the follow-up was observed. All studies evaluated growth at different time points (six weeks to five years). In one study, magnetic resonance imaging (MRI) was used to assess adipose tissue content and distribution at the age of six months [73]. Four studies assessed the long-term risk of diabetes [71,72,75,76].

## 4. Discussion

The growth patterns of breastfed SGA infants, as shown in the few existing papers analyzed in this review, seem to lead to some conclusions that challenge conventional expectations about early nutrition and development. It has been demonstrated that infants who are breastfed exhibit a greater rate of weight gain during the initial two to three months following birth, when compared to those who are formula-fed. However, this is inverted for the remainder of the infant period, and breastfeeding has been associated with a reduced rate of weight gain [77,78,79].

Preterm SGA neonates present a complex clinical challenge due to their increased nutritional needs. In a cohort of preterm neonates, Belfort et al. reported that SGA neonates fed on human milk exhibited a slower weight gain compared to those fed on formula until hospital discharge. However, the velocity of weight gain in SGA human milk-fed neonates was higher than that observed in both human milk-fed and formula-fed AGA neonates [66]. Hofi et al. also reported lower z-scores in SGA preterm neonates who were fed human milk compared to those who were fed formula at hospital discharge. At the corrected age of two years, the rate of weight catch-up did not differ significantly between the two groups; however, the head circumference catch-up was achieved in only 43% of children who were breastfed and 71% of those who were formula-fed (*p* = 0.05) [57]. This creates an apparent paradox where the feeding strategy, appearing suboptimal in the short term, may confer superior long-term advantages [80]. Studies have demonstrated that breastfeeding is associated with improved neurodevelopmental outcomes in preterm neonates despite initial suboptimal growth [80,81]. Nevertheless, in a cohort of full-term SGA infants, Santiago et al. found that exclusively breastfed infants showed an accelerated weight gain in the first six months compared to their peers but did not develop overweight or obesity later. Indeed, these infants remained leaner at preschool age, with weight gain mainly from lean muscle, indicating breast milk may influence growth patterns differently than formula [81].

Breastfeeding facilitates sophisticated biological programming, optimizing long-term metabolic health through moderated weight gain patterns. Breastfed infants exhibit a slower weight gain after the age of two to three months, resulting in lower weight z-scores but superior body composition, with reduced fat mass and enhanced lean tissue development [82,83,84]. This growth pattern is of particular significance in SGA infants. It has been demonstrated that breastfeeding facilitates initial catch-up growth. Subsequently, the transition to a more sustained pattern of growth has been shown to reduce the risk of obesity [71,82,85]. Furthermore, it has been observed that SGA infants exhibit a heightened preference for calorie-dense foods. It is hypothesized that breastfeeding’s self-regulatory mechanisms may offer biological constraints against the development of excessive intake patterns, which can lead to pathological catch-up growth [86,87,88].

The fundamental distinction between direct breastfeeding and alternative feeding approaches lies in the infant’s capacity for autonomous appetite regulation, which prioritizes metabolic health over maximum weight velocity [79,89]. SGA infants demonstrate altered appetite regulation, including impaired satiety signaling, and enhanced orexigenic responses [90]. Infant-controlled feeding enables them to achieve appropriate catch-up growth through self-regulation. This is in contrast to the externally imposed schedules of formula feeding. Breastfeeding provides a protective framework accommodating these appetite irregularities, while preventing the metabolic consequences associated with uncontrolled growth acceleration [79,89,91].

The metabolic benefits of breastfeeding extend beyond mere anthropometric measures, encompassing the programming of protective hormones. A study of breastfed SGA infants revealed that they exhibited healthier hormone levels, particularly glucagon-like peptide-1 (GLP-1), which plays a pivotal role in regulating long-term blood sugar control [72]. Moreover, Gupta et al., in a randomized study that compared the risk of early hyperinsulinemia and insulin resistance in full-term, low birth weight neonates that received either human milk exclusively or fortified human milk, reported a lower risk in exclusively human milk-fed infants [76]. Visuthranukul et al. observed a similar risk of insulin resistance in a cohort of preterm SGA infants fed human milk compared to AGA infants [65]. While formula-fed SGA infants often exhibit concerning elevations in growth hormones and fat-regulating proteins, their breastfed counterparts maintain more physiological levels [75]. Santiago et al., in a longitudinal study, reported normalized blood pressure, glucose, HOMA-IR, and insulin levels at preschool age in breastfed SGA children, suggesting that the metabolic disadvantage associated with their birth status was effectively eliminated [71]. Furthermore, a potential protective effect against diabetes, hypertension, and cardiovascular disease has been suggested [75]. It has been demonstrated that human milk has the potential to mitigate the risk of subsequent obesity effectively [71,82,85]. Li et al. assessed the growth patterns of 296 SGA neonates until the age of five years. The authors concluded that the rates of excessive weight catch-up at two years were lower in human milk-fed infants and that this was associated with a lower risk of obesity at the age of five years [74].

The metabolic programming advantages of breastfeeding in SGA neonates extend beyond nutritional provision to encompass the biological communication between maternal physiology and infant developmental needs. Human milk contains bioactive compounds, including oligosaccharides, lactoferrin, immunoglobulins, cytokines, and growth factors, that orchestrate infant metabolism, immune development, and growth patterns [32,92,93]. Moreover, the establishment of the infant intestinal microbiome through milk-derived communities influences both immune maturation and metabolic regulation [94,95]. The temporal variation in milk composition, with peak bioactive concentrations in colostrum followed by continued adaptation, suggests there is evolutionary programming responding to changing infant requirements [31,32,33,34]. For SGA infants, this adaptive capacity becomes critical, as their metabolic needs may deviate from population norms, necessitating individualized biochemical signaling that only fresh maternal milk may provide [96,97].

To provide adequate clinical support for breastfeeding in SGA neonates, it may be necessary to reconsider the growth monitoring standards currently employed. The implementation of breastfeeding paradigms has encountered significant clinical resistance from growth-focused monitoring systems developed for formula-fed populations. Traditional growth references derived from formula-fed populations fail to recognize breastfed infants’ distinct trajectory of rapid initial growth followed by naturally moderated weight gain [98]. This pattern frequently triggers unnecessary interventions, disrupting beneficial feeding relationships, that are particularly problematic for SGA infants whose growth may deviate further from norms [99]. However, more recent growth charts better represent breastfed infants, and there is evidence that lower percentile expectations increase breastfeeding rates [100,101,102,103]. Nevertheless, slower growth trajectories in breastfed SGA infants may represent optimal metabolic programming rather than nutritional inadequacy, demanding clinical protocols that distinguish adaptive patterns from pathological failure while optimizing long-term outcomes for this vulnerable population [82,104].

The findings of the reviewed studies suggest that traditional neonatal nutrition approaches for preterm SGA infants might require reconsideration. Data supports prioritizing exclusive human milk feeding, while accepting a slower initial growth as potentially beneficial, rather than pursuing aggressive growth targets through formula supplementation. This speculation demands support by appropriate monitoring protocols distinguishing the appropriate slower growth from pathological failure [105,106]. However, further research on optimal human milk fortification strategies that maximize both short-term growth and long-term metabolic outcomes is needed [41]. Family counseling becomes critical, as parents often experience significant growth-related anxiety. Healthcare providers must communicate that slower initial growth represents an adaptive advantage rather than nutritional compromise, while maintaining vigilance for genuine growth failure [107].

Despite these encouraging findings, several methodological limitations must be acknowledged. The heterogeneity in study designs, varying definitions of exclusive human milk feeding, the inclusion or exclusion of neonates that received donor milk when maternal milk was unavailable, and differences in fortification strategies across studies complicate direct comparisons. Moreover, the observational nature of many studies introduces potential confounding variables, including maternal factors, socioeconomic status, and varying clinical practices that may influence outcomes beyond feeding type alone. The relatively small effect sizes observed in some studies raise questions about clinical significance. Additionally, the generalizability of findings from highly controlled research settings to routine clinical practice remains uncertain. Nevertheless, the consistency of metabolic benefits across multiple investigations may suggest protective mechanisms underlying human milk feeding approaches.

## 5. Conclusions

The present literature suggests that breast milk may have a beneficial effect on the growth patterns of both preterm and full-term SGA infants. Despite the limited number of studies and the presence of several limitations, as previously discussed, the findings demonstrated consistency across the gestational age and postnatal age groups. Nutrition and growth in SGA infants and children present a particular challenge for clinicians, as these patients have special nutritional needs and growth patterns. Healthcare providers should be capable of distinguishing between standard adaptive growth and pathological failure. The long-term metabolic benefits of human milk are well-established, and these are especially valuable when considered within the context of the SGA population, which is known to be at risk of obesity and cardiometabolic consequences. However, the paucity of research regarding human milk feeding in SGA populations poses a significant dilemma for healthcare providers, who must judiciously balance long-term benefits against immediate growth concerns. Future larger prospective studies are needed to evaluate human milk feeding and human milk fortification in association with growth and long-term outcomes in SGA infants.

## Figures and Tables

**Table 1:** Studies evaluating the growth outcome of human milk-fed SGA preterm infants.

Study	Type of Study	Population	Study Groups	Gestational Age	Duration of Breastfeeding	Mother’s Own Milk or Donor Milk	Type of Fortification	Type of Measurements	Growth Findings	Other Findings
Fleig et al., 2021 [64]	Multicenter retrospective cohort	420 SGA neonates, BW < 1250 g	197 CM, 223 HM	28 weeks (median)	Hospital discharge	MOM	CMF	Weight, HC, weight gain velocity, and head growth rate	Improved z-scores	Reduction in NEC, surgical NEC, and LOS
Visuthranukul et al., 2019 [65]	Single-center, longitudinal cohort study	51 preterm neonates, BW < 1250 g	33 AGA HM, 18 SGA HM	26.4 weeks (AGA), 29.3 weeks (SGA)	34 weeks CA	ND	ND	Anthropometric measurements, serum glucose, non-fasting insulin (12–15 months CA), and X-ray absorptiometry (18–22 months CA)	SGA neonates demonstrated greater catch-up growth without increased adiposity	No increased risk of insulin resistance compared to AGA
Belford et al., 2019 [66]	Multicenter observational study	138,703, GA < 32 weeks	8977 HM, 65,706 HM and CM/HM and FHM, and 64,020 CM	29 weeks (HM), 29 weeks (HM and CM/HM and HMF), and 28 weeks CM	Hospital discharge	ND	ND	Weight, HC, weight gain velocity, and head growth rate	Unfortified HM nutrition was associated with lower weight and HC	SGA neonates had higher absolute weight gain and head circumference growth compared to AGA
Hofi et al., 2022 [67]	Retrospective cohort	80 SGA preterm	40 HM, 40 CM	33.8 weeks (HM), 34.7 weeks (CM)	ND	MOM	ND	Weight, length/height, and HC	HM was associated with a twofold loss in weight and length z-scores compared to CM; CM was associated with a fourfold increase in HC z-scores compared to HM	At 2 years CA, both groups had similar positive change in weight and HC z-scores; weight catch-up: 69% HM vs. 86% CM (*p* = 0.10); height catch-up: 40% HM vs. 68% CM (*p* = 0.02); HC catch-up: HM: 43% vσ; and PF: 71%, (*p* = 0.05)
Bushati et al., 2021 [68]	Prospective observational with historical control	64 preterm, BW < 1000 g	15 HM (40% SGA), 49 FHM (18.4% SGA)	28 weeks (HM), 26 weeks (FHM)	HM group: Transition to FHM after 34 weeks CA	MOM or donor HM	CMF	Tolerability of HM compared to FHM; assessment of growth parameters	Better tolerability of HM (only when unadjusted for SGA status); HM group had significantly lower discharge z-scores in weight and length compared to FHM	Nutrition with HM offered no benefit on the incidence of NEC, LOS, and parenteral nutrition days compared to FHM
Vesel et al., 2023 [69]	Multicenter prospective observational cohort study	1114 neonates, BW 1500–2000 g			Variable	ND	ND	Feeding practices, birthweight at 2 weeks and 6 months	Preterm SGA infants had 1.89 and 2.32 times greater risks of being stunted and underweight at 6 months compared to preterm AGA; full-term SGA infants had 2.33, 2.89, and 1.99 times higher risks of being stunted, underweight, and wasted compared with preterm AGA	SGA status (full-term or preterm) and lack of birth weight regain by 2 weeks are important risk parameters for growth failure
Vizzari et al., 2023 [70]	Retrospective study	175 SGA neonates, GA 34–36 weeks	18% HM, 36% HM and CM, and 46% CM	35.2	ND	MOM	CMF	Weight, length/height, and HC	Infants receiving any HM at discharge had a lower risk of failing to achieve catch-up growth in weight and length at 36 months	Growth trajectory during early infancy and catch-up growth during the first year could be affected by different variables such as being born singleton, having IUGR, and being breastfed

SGA: small for gestational age; BW: birth weight; CM: cow milk; HM: human milk; MOM: mother’s own milk; CMF: cow milk fortifier; HC: head circumference; NEC: necrotizing enterocolitis; LOS: late onset sepsis; AGA: appropriate for gestational age; CA: corrected age; GA: gestational age; FHM: fortified human milk; IUGR: intrauterine growth restriction; and ND: no data.

**Table 2:** Studies evaluating the growth outcome of human milk-fed SGA full-term infants.

Study	Type of Study	Population	Study Groups	Duration of Breastfeeding	Duration of Follow-Up	Objective of the Study	Type of Measurements	Growth Findings	Further Findings
Santiago et al., 2020 [71]	Prospective cohort study	32 neonates	20 SGA, 12 AGA	180 days	Until preschool age	Evaluation of the cardiometabolic profile of SGA infants and comparison to that of AGA	Weight, height, head, neck and waist circumference, skinfolds, fasting blood glucose, insulin, HOMA-IR, and blood pressure (at preschool age)	85% of SGA infants had recovery of anthropometric parameters for age within the first six months; weight gain velocity was significantly higher than that of AGAs (*p* < 0.001); and no overweight/obese	Similar cardiometabolic risk factors at preschool AGA and SGA, potentially due to the protective effect of exclusive breastfeeding
Díaz et al., 2015 [72]	Retrospective cohort study	117 neonates	63 AGA HM, 28 SG HM, and 26 CM	ND	Until 4 months	Evaluation of the circulating concentrations of GLP-1 in the study groups	Auxological assessments (at birth, 2 weeks, and 4 months) of GLP-1 levels (birth, 4 months)	Breastfeeding positively affects weight and length	Lower long-term risk of diabetes by preserving normal GLP-1 levels
Modi et al., 2006 [73]	Prospective cohort study	35 neonates	25 AGA, 10 growth-restricted	6 weeks	Until 6 weeks	Assessment of the adipose tissue content and distribution (birth, 6 weeks) in relation to intrauterine growth restriction, extrauterine growth, and infant nutrition	MRI (to assess adipose tissue content and distribution)	SGA infants exhibited complete catch-up in head growth and adiposity by six weeks	Lower risk of obesity in exclusively breastfed infants
Li et al., 2022 [74]	Prospective cohort study	296 SGA		ND	Until 5 years	Evaluation of the utility of early postnatal growth of SGA infants as a predictor for later obesity	Weight, length, and height	Excessive catch-up rates were lower in exclusively breastfed infants; breastfeeding promotes optimal growth	Excessive catch-up weight growth in SGA infants aged 0–2 years increases the risk of obesity at 2–5 years of age
Zegher et al., 2012 [75]	Prospective cohort study	176 neonates	72 AGA HM, 46 HM SGA, and 56 CM SGA	4 months	4 months	Evaluation of the effects of HM vs. CM on weight partitioning and endocrine state of SGA infants	Body composition assessment, high-molecular-weight adiponectin, and IGF-I (birth, 4 months)	Lean mass recovery over fat mass	Normal levels of high-molecular-weight adiponectin, IGF I in SGA HM, and elevated in SGA CM
Gupta et al., 2010 [76]	Randomized study	52 neonates, BW < 2500 g	26 SGA HM, 26 SGA FHM	3 months	Until 3 months		Weight, length, height (every 15 days until 3 months), and 4 h fasting glucose and insulin levels (birth, 3 months)	Slower and steadier weight gain in HM compared to FHM	Reduced risk of early hyperinsulinemia and insulin resistance in HM (lower 4 h fasting glucose and insulin levels in HM group compared to FHM)

SGA: small for gestational age; AGA: appropriate for gestational age; HOMA-IR: homeostasis model assessment of insulin resistance; CM: cow milk; HM: human milk; GLP-1: glucagon-like peptide-1; MRI: magnetic resonance imaging; IGF-1: insulin growth factor-1; FHM: fortified human milk; BW: birth weight; and ND: no data.
